# Wire Rope Defect Recognition Method Based on MFL Signal Analysis and 1D-CNNs

**DOI:** 10.3390/s23073366

**Published:** 2023-03-23

**Authors:** Shiwei Liu, Muchao Chen

**Affiliations:** 1 College of Engineering, Huazhong Agricultural University, Wuhan 430070, China; 2 Network Information and Modern Education Technology Center, Guangdong Communication Polytechnic, Guangzhou 510650, China; 3 School of Computer Science, Wuhan University, Wuhan 430072, China

**Keywords:** defect detection, signal analysis, convolutional neural network (CNN), feature extraction, wire rope

## Abstract

The quantitative defect detection of wire rope is crucial to guarantee safety in various application scenes, and sophisticated inspection conditions usually lead to the accurate testing of difficulties and challenges. Thus, a magnetic flux leakage (MFL) signal analysis and convolutional neural networks (CNNs)-based wire rope defect recognition method was proposed to solve this challenge. Typical wire rope defect inspection data obtained from one-dimensional (1D) MFL testing were first analyzed both in time and frequency domains. After the signal denoising through a new combination of Haar wavelet transform and differentiated operation and signal preprocessing by normalization, ten main features were used in the datasets, and then the principles of the proposed MFL and 1D-CNNs-based wire rope defect classifications were presented. Finally, the performance of the novel method was evaluated and compared with six machine learning methods and related algorithms, which demonstrated that the proposed method featured the highest testing accuracy (>98%) and was valid and feasible for the quantitative and accurate detection of broken wire defects. Additionally, the considerable application potential as well as the limitations of the proposed methods, and future work, were discussed.

## 1. Introduction

Wire rope is one of the most frequently used loading and bearing tools in various engineering applications, such as elevators, cableways, bridges, cranes, and hoisting equipment, and plays a significant role in guaranteeing human life and property [1,2]. However, plenty of defects regularly occur in the in-service steel wire rope owing to damage and failure both in strength and structure, which may cause severe accidents and even fatalities. Such defects include the fatigue fracture of individual wire due to repeated friction and complex state-of-stress, fretting, and formation of frictional martensite, which is often the precursor of wire breakage and even the fusion of individual wire nearby due to local frictional heating [3,4,5]. Generally, these defects can be classified into two types, namely, local faults (LF) such as broken and cracked wires, and the loss of metallic sectional areas (LMA), such as wear and abrasion. According to the wire rope testing and discard criteria, when the electromagnetic testing apparatus and methods are applied to the wire rope, a certain number of broken wires or sectional area losses are unacceptable [6]. Dalvir Kaur et al. [7] presented the characterizations of LF and LMA through a hall sensor signal analysis and a comparison from the perspective of feature extraction, correlation, energy, and a homogeneity investigation. S.K. Kashyap et al. [8] reviewed the steel wire rope discard criteria from different countries and testing methodologies as well as the inspection instruments, which indicated that quantitative inspection by suitable testing methods [9,10] and apparatus [11] is the key to determining the feasibility of a wire rope. To improve the signal processing and defect inspection performance, except for the common magnetic sensors including magnetic flux gate transducer [12], hall [13], induction coil, and the magnetic resistive sensors of GMR [14], AMR and TMR [15,16], various magnetic bridge circuit-based sensors were proposed, such as the three-dimensional MFL sensor [17], tunnel magnetoresistive-based circular MFL sensor [18], radial magnetic concentrator [19], parallel [20] as well as the open magnetizer based sensor [21], which behave better than common magnetic sensors in generating wire rope inspection signals and defect recognition. Aiming at the challenges of weak wire rope signal processing [22,23], some magnetic sensors based on new nondestructive testing principles were also reported, for instance, the magnetic focusing sensor [24]. However, most of these sensors have difficulty in balancing the signal recognition accuracy and defect detection types.

Due to the complex wire rope testing environments and monitoring conditions, wire rope detection signals are usually mixed with various interferences and noises in MFL testing, such as electromagnetic background noise, and strand and swing signals, which make the real defect signals difficult to distinguish. Thus, many signal denoising methods, such as the wavelet transform, empirical mode decomposition, blind source separation, and multifarious filtering techniques, have been reported. Peter W. Tse et al. [25] proposed a short-time Fourier transform (STFT) and wavelet combined signal analysis method for steel wire rope testing by ultrasonic guided wave, which could identify the defect location and severity successfully. Tian Jie et al. [26] applied the mathematical morphological theory and non-sampled wavelet filtering methods with online detection for mine wire rope, which filtered the baseline drift noise and improved the signal-to-noise ratio (SNR) to 10 dB. Owing to the development of image-processing techniques, various image signal-processing and machine learning methods are also introduced to steel wire rope defect inspection and recognition. For instance, Donglai Zhang et al. [27] designed a hall sensor array to capture the magnetic flux leakage (MFL) image and ultilized the gray level co-occurrence matrix to extract typical features as the input of back propagation (BP) network, which showed good performance in the quantitative recognition of different wire rope defects. Qinghua Mao et al. [28] presented an improved decision tree support vector machine (SVM) algorithm, and the classification accuracy for steel cord conveyor belt defects was verified by particle swarm optimization and related experiments. Other machine learning algorithms including the k-nearest neighbor (KNN), artificial neural network (ANN), and logistic regression as well as variational mode decomposition [29,30,31] are also applied in broken wire classification, which exhibit high sensitivity and detection accuracy in experiments. Xiaoguang Zhang et al. [32] verified a new algorithm called the variable step incremental extreme learning machine (ELM), which featured a faster classification speed and higher classification accuracy for different broken wires of steel wire rope. Esther-Sabrina Wacker et al. [33] achieved a robust localization of wire rope surface defects by means of an anomaly detection algorithm, which was immune to the illumination setting and the reflectance properties of the materials, and the defect detection accuracy could reach 95%.

As the MFL imaging and deep learning techniques further applied to wire rope defect inspection, many computer vision-based feature extraction and fusion methods have been proposed [34]. Nevertheless, most of these inspection methods and algorithms are only limited to surface defect detection, and the inner damage as well as the stress concentration is usually omitted, which may cause potential safety hazards in engineering applications. As the requirements of high accuracy defect inspection are desperately needed, deep learning and deep neural networks are also gradually being applied to wire rope-testing signal-processing and data analysis, especially with regard to convolutional neural networks (CNNs) [35] which have a significant impact on all professions. Composed of an input layer, convolutional layer, pooling layer, and fully connected layer, CNNs have been widely used in two-dimensional (2D) data processing for medical imaging, face recognition, text classification and one- dimensional (1D) voice-signal filtering, three-dimensional (3D) scene reconstruction, as well as the video signal recognition and semantic segmentation. A lot of research can be found in related application fields, such as in breast, skin, lung and brain cancer inspection image classification using CNNs in the medical application proposed by Li Chen [36], Qing Li [37], Titus Josef Brinker, MD et al. [38], which can achieve automatic and efficient image classification and complete the task of medical diagnosis. Ueli Meier et al. [39] reported a CNN-based handwritten character classification method through 78,125 different 7-net committees applied to NIST SD 19, which dropped the error rate to 0.27%. Typical applications of CNNs may also include 1D electrocardiograph (ECG) signal classification [40], large-scale video [41], gas [42], cell as well as tstar galaxy classification and recognition [43,44]. As regards steel wire rope, Gongbo Zhou et al. [45] applied CNNs to the health monitoring of balancing tail ropes serviced in a hoisting system, which could achieve fault detection including disproportional spacing, twisted rope, broken strand and wires automatically in real time. Zhiliang Liu et al. [46] proposed a CNN-based surface defect detection method for wire rope, which was proved to have powerful learning ability and could extract discriminant features automatically. Ping Zhou et al. [47] proposed an automatic CNN-based wire rope surface damage detection method, which was also demonstrated to have a high degree of diagnostic accuracy through a large number of tests and contrast analyses.

Although the learning ability and recognition accuracy have rapidly improved, and the modification results for various CNN-based architectures were also presented [48], the quantitative and online wire rope MFL-testing effectiveness and efficiency for both inner and surface defects are still limited, while the computer vision-based 2D image processing and CNN methods can only detect surface defects, which all make wire rope defect recognition very challenging. Consequently, a wire rope defect signal-processing and CNN-classification combined method was proposed for 1D magnetic flux leakage testing (MFL) in this study. Wire rope defect detecting principles and preliminary data analysis from the perspective of time-frequency domain and statistical properties are presented in Section 2. The detailed signal denoising methods by wavelet transform and feature extraction techniques are described in Section 3. The proposed CNN-based algorithms for wire rope defect recognition and comparisons results with conventional machine learning methods are explained in Section 4. Finally, the advantages and shortcomings, as well as the future work regarding the combined methods applied to wire rope inspection, are summarized and discussed in Section 5.

## 2. Wire Rope Defect Signal Data Analysis

**Figure 1:**
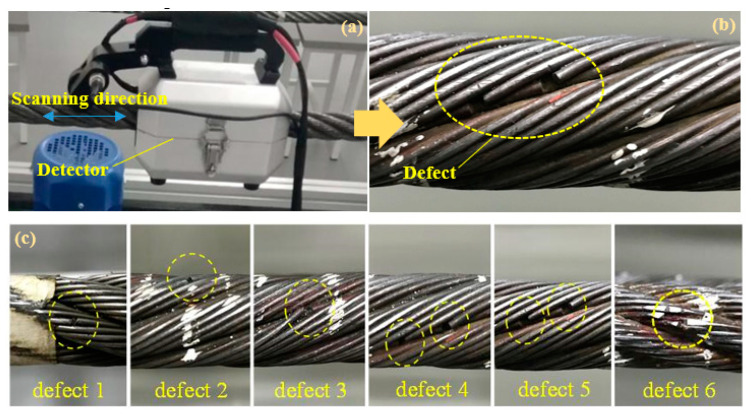
Wire rope testing devices and experimental defects: (**a**) wire rope detector; (**b**) wire rope defect; and (**c**) typical defect 1 to defect 6.

The wire rope detection signals are mainly obtained through the magnetic flux leakage (MFL) testing devices shown in Figure 1a, when the detector containing a group of magnetic sensor arrays and a magnetic excitation apparatus is scanned along the tested steel wire rope with a diameter of 29 mm and testing speed of 20 cm/s. The magnetic flux will leak from the position of wire rope discontinuities or defects, including broken wires, abrasion and wear, which will simultaneously be captured by the magnetic sensitive element of the inductive coil circumferentially installed inside the detector inner wall. Thus the leaked magnetic signals can be converted to electrical signals and transferred to data acquisition modules. A typical wire broken defect is expressed in Figure 1b, where two neighboring broken wires with a single wire diameter of 1.8 mm are appeared. In addition, six groups of wire broken defects are presented in Figure 1c. Specifically, defect 1 is a single broken wire near the groove of the strand, defect 2 is a single broken wire on the surface of the strand convex, defect 3 is a curled broken wire, defect 4 and defect 5 are composed of two discontinuous broken wires located in different positions near the strand groove, defect 6 represents a discontinuous broken wire on the convex position of the strand.

**Figure 2:**
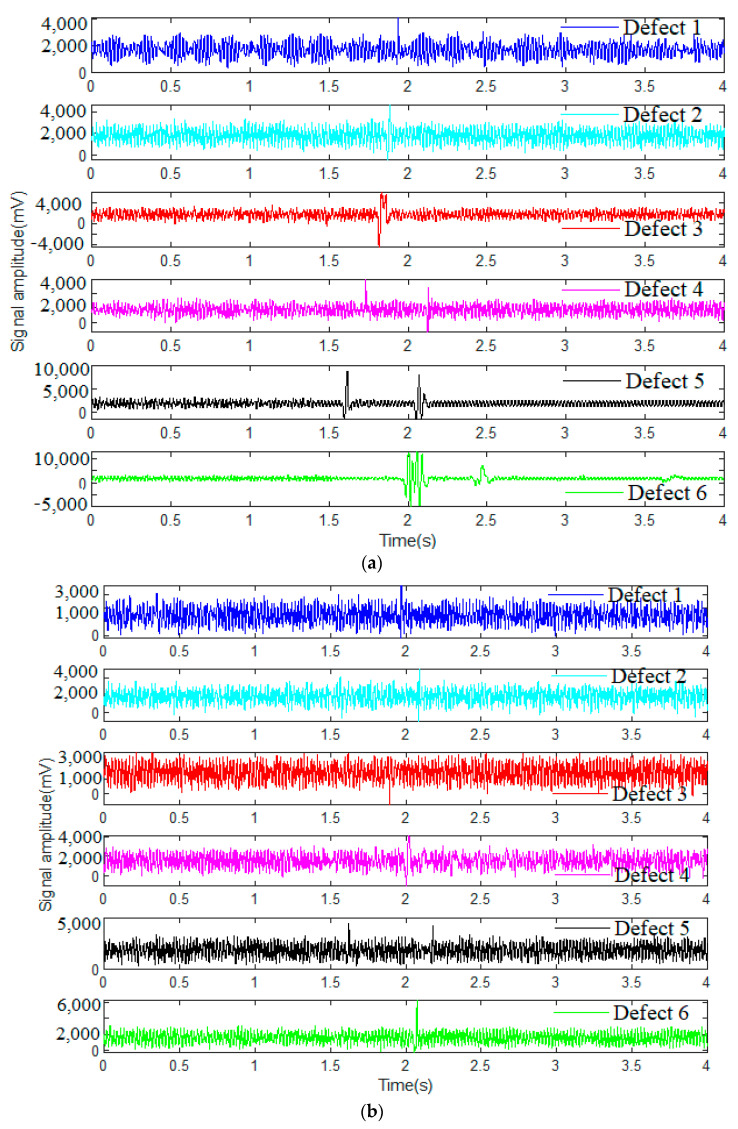

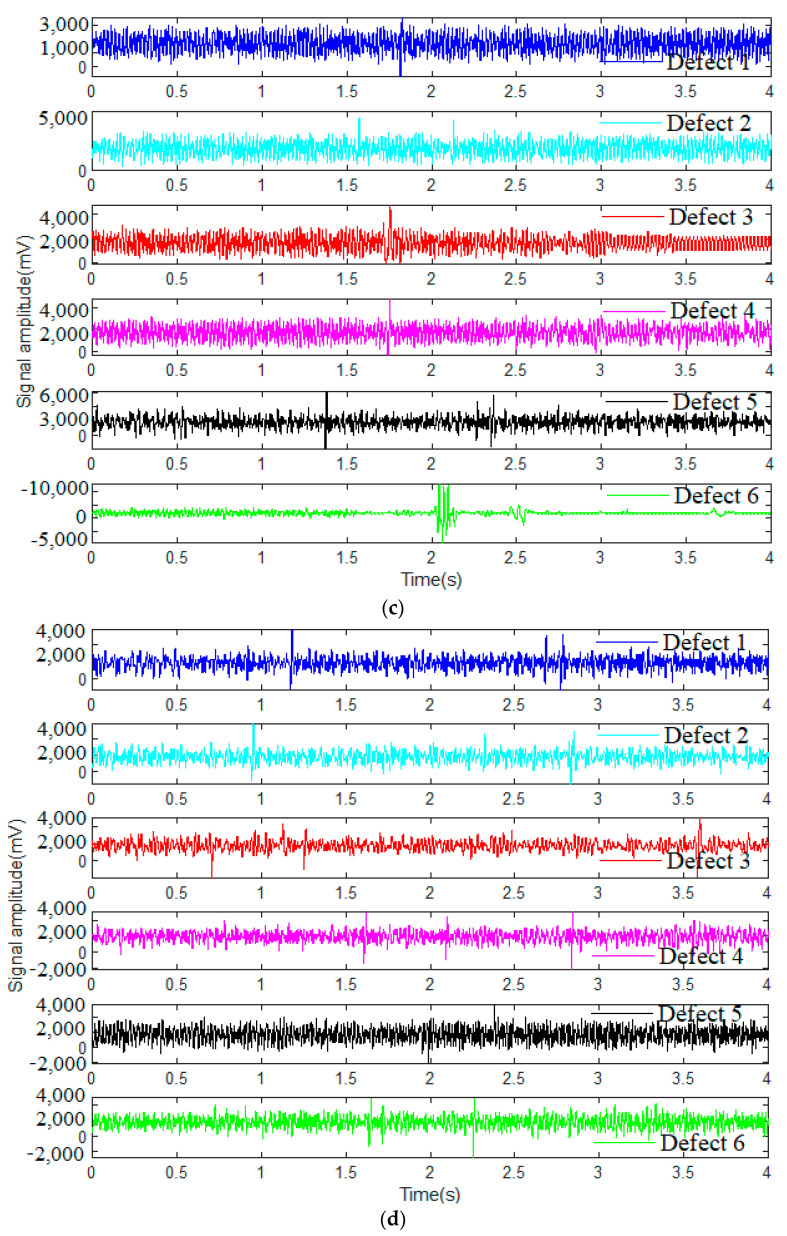
Wire rope detection signals from different datasets: (**a**) wire rope detection signal of dataset 1; (**b**) wire rope detection signal of dataset 2; (**c**) wire rope detection signal of dataset 3; and (**d**) wire rope detection signal of dataset 4.

Consequently, the steel wire rope defect signals are obtained from the signal monitoring software installed on the computer. Four groups of wire rope detection signals tested under different strong swing, vibration and surrounding electromagnetic interference noise conditions are illustrated in Figure 2, and there are six main defects in each of these datasets, which are labeled as defect 1 to defect 6. Obviously, all the original signals of wire rope testing data are featured with interferences of strand and swing signals as well as various background noises. However, the defect types of the wire rope can hardly be distinguished just by the signal characteristics in the time domain, especially when the detection conditions are hostile and the defect is tiny with weak characterizations. Typical signals from different datasets are shown in Figure 2b–d.

From the perspective of frequency domain analysis, a Fast Fourier Transform (FFT) is conducted for the wire rope testing signals. The discrete wire rope detecting signals can be defined as a signal series of *s*(*n*) with the length of *N*, namely, *n* = 0, 1, 2, …, *N* − 1, and the Fourier transform is described as,

(1) $$S(k)=\sum_{n=0}^{N-1}s(n)\begin{array}{cc} e^{-j\frac{2kn\pi }{N}} & k=0,1,\ldots ,N-1 \end{array}$$

**Figure 3:**
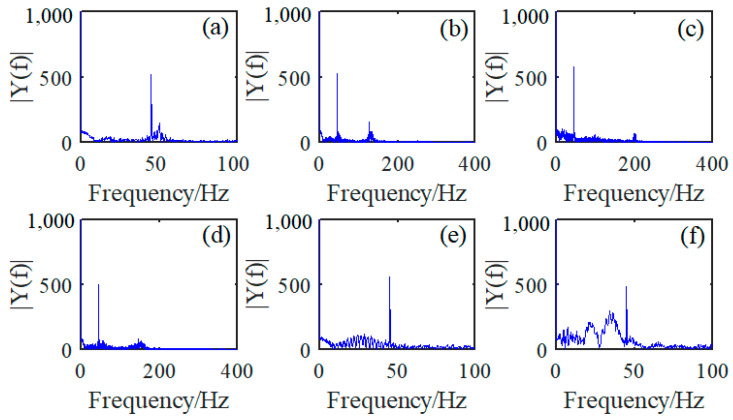
FFT of different wire rope defect signals: (**a**) FFT from defect 1; (**b**) FFT from defect 2; (**c**) FFT from defect 3; (**d**) FFT from defect 4; (**e**) FFT from defect 5; and (**f**) FFT from defect 6.

The typical FFT calculating result for six defect signals mentioned in dataset 1 is obtained and expressed in Figure 3. Specifically, defect signals FFT computed for defect 1 to defect 6 are presented in Figure 3a–f, respectively. Apparently, the main frequency bands of defect signal 1 and signal 5 shown in Figure 3a,e are distributed around the frequency of 45 Hz, while the defect signals 2, 3 and 4 presented in Figure 3b–d are all accompanied with regular noises and extended frequency components around 130 Hz, 200 Hz and 150 Hz. In addition, defect signals from defect 5 and 6 expressed in Figure 3e,f are both featured with a main frequency of 45 Hz and an extended interference signal frequency band from 0 to 40 Hz. However, almost all these FFT calculation results from dataset 1 are featured with a common and prominent frequency around 50 Hz. 

**Figure 4:**
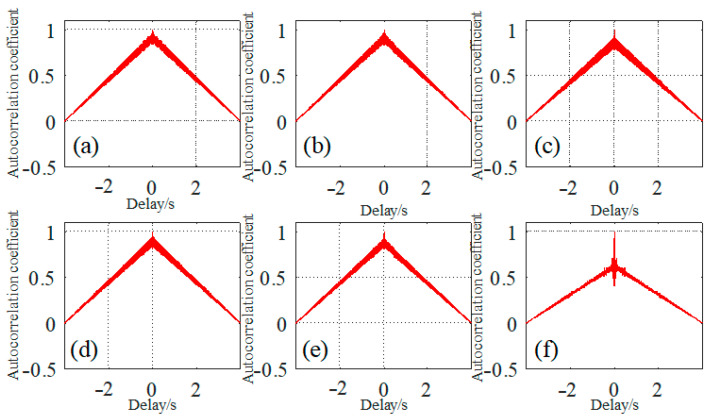
Autocorrelation analysis for different wire rope defect signals: (**a**) for signals from defect 1; (**b**) for signals from defect 2; (**c**) for signals from defect 3; (**d**) for signals from defect 4; (**e**) for signals from defect 5; and (**f**) for signals from defect 6.

The typical signal analysis in frequency domain manifests that although the defect signals are mainly characterized with a fixed frequency around 50 Hz, various interferences of noises, such as the wire rope strand, swing signals and electromagnetic background signals, are inevitable. As for the frequency components distributed in different locations in the FFT diagram, it makes the accurate evaluation and recognition of different steel wire rope defects very challenging. Furthermore, to investigate the interdependence of these time series signals, an autocorrelation analysis is also conducted. The autocorrelation coefficient of *R_k_* can be defined as,

(2) $$R_{k}=\frac{\sum_{i=1}^{n-k}\left(s_{i}-\bar{s})(s_{i+k}-\bar{s}\right)}{\sum_{i-1}^{n}{\left(s_{i}-\bar{s}\right)}^{2}}$$

where the *S_i_* is the time series signal of wire rope inspection, *s* is the mean value of the signal, and the detailed autocorrelation function are depicted in Figure 4. The typical autocorrelation coefficients for six different wire rope defect signals from dataset 1 and mentioned in Figure 2a are separately expressed in Figure 4a–f, which indicates that as the absolute delay time increases, the correlation between the front and back time series of the testing signal fades off. When the delay time is zero, all the autocorrelation coefficients are approximate to 1 except the time series of defect 6 shown in Figure 4f which may be caused by the regular noises.

**Figure 5:**
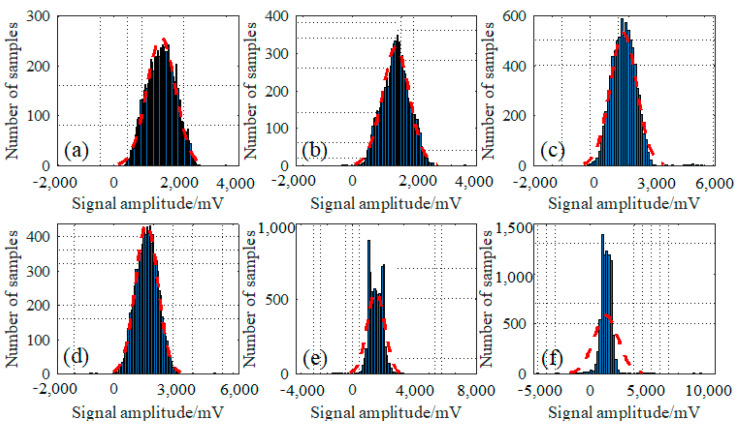
Probability density distribution of different wire rope defect signals: (**a**) for signals from defect 1; (**b**) for signals from defect 2; (**c**) for signals from defect 3; (**d**) for signals from defect 4; (**e**) for signals from defect 5; and (**f**) for signals from defect 6.

Additionally, the statistical characteristics for the detecting signals of these typical wire rope defects from the perspective of probability density distribution analysis were also conducted and presented in Figure 5.

Similarly, the statistical distribution for defect 1 to defect 6 are separately presented in Figure 5a–f, especially indicated by the red dotted lines. According to the fitting curves expressed in red line, it can be deduced that all these defect signals are distributed in an approximate normal distribution. In other words, most of these time series signals of defects are distributed around the median of 2000 mV, and the numbers of signal points distributed around the two sides of the mid value decrease gradually as the signal amplitude increases or decreases. Notably, the signals of defect 5 and defect 6 shown in Figure 5e,f are characterized with some higher unique points, which may be caused by their larger defect signal amplitudes. What is more, the testing signals from defect 5 and defect 6 are distributed more compactly, judged by the horizontal axis of the signal amplitude compared with the other defect types shown in Figure 5a–d, which provides a reference to the feature extraction for signal and defect classification.

**Figure 6:**
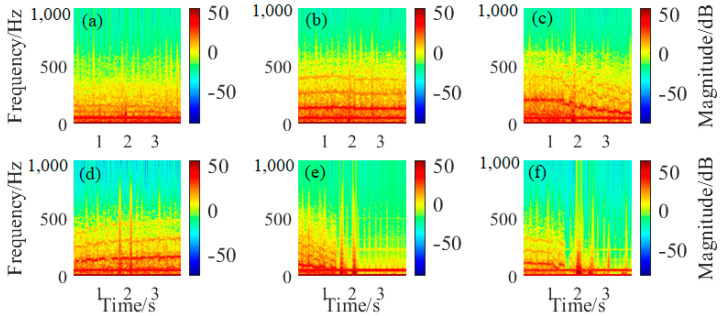
Short time Fourier transform (STFT) for different wire rope defect signals: (**a**) for signals from defect 1; (**b**) for signals from defect 2; (**c**) for signals from defect 3; (**d**) for signals from defect 4; (**e**) for signals from defect 5; and (**f**) for signals from defect 6.

To investigate different wire rope defect signals more comprehensively, a time-frequency joint analysis method by STFT was also considered. The typical defect signals from dataset 1 by STFT can be described by the following formula,

(3) $$S_{s}(m,n)=\sum_{k=-\infty }^{\infty }s(k)\cdot g(k-m)e^{-j2\pi nk}$$

where *s*(*k*) is the original time series of wire rope defect signal, *g*(*k*) is the window function. When the moving function by the Hamming window is applied and an overlap of 50% is set, the STFT for six different defect signals as mentioned above are presented in Figure 6a–f, respectively.

**Figure 7:**
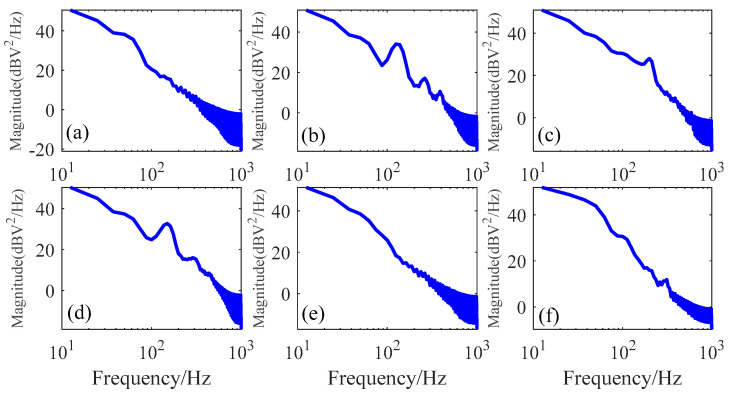
Power spectral density (PSD) of different defect signals: (**a**) for signals from defect 1; (**b**) for signals from defect 2; (**c**) for signals from defect 3; (**d**) for signals from defect 4; (**e**) for signals from defect 5; and (**f**) for signals from defect 6.

As can be seen by the STFT results, it can be observed that almost all these defect signals are distributed within the frequency band smaller than 400 Hz, and the signal with the low frequency component of 50 Hz exists throughout all the sampling process especially in Figure 6a–c. In addition, there are two apparent signal enhancement regions between the time intervals of 1.5 s to 2.5 s in Figure 6d,e, which is exactly consistent with the original signal analysis for defect 4 and defect 5 in dataset 1. Similarly, the continuous signal enhancement area throughout the time intervals of 2 s to 3.5 s in the STFT spectrum in Figure 6f not only demonstrates the signal distribution characterizations in the time and frequency domains, but also verifies the FFT analysis mentioned in Figure 3, which further increases the resolution of signal components analysis. On the other hand, the power spectral density (PSD) estimation by Welch’s overlapped segment averaging estimator was also conducted, and the PSD was mainly calculated according to the follow formula,

(4) $$P(\omega )=\underset{T\to +\infty }{\lim}\frac{{\left|F(\omega )\right|}^{2}}{2\pi T}$$

where *F*(*ω*) is the Fourier transform for the wire rope detection signal, and the period of T can be viewed as the limit of infinity for the discrete signal. The detailed PSD analysis for six typical wire rope defect inspection signals from dataset 1 is illustrated in Figure 7.

According to the PSD results, we can conclude that these signals are mainly distributed in low frequency band, where the amplitude of PSD is higher than that in the high frequency region. That is to say, as the signal frequency increases, the signal energy decreases gradually and there still exist many noise components in the high frequency region observed by the oscillation waveforms. Most importantly, a prominent raised waveform in the frequency around 100 Hz can be found in Figure 7b–d, which is also an indication of the wire rope defect signal and can be viewed as a reflection of the extended frequency components explained in Figure 3b–d.

## 3. Signal Denoising and Feature Extraction

To build the training and testing datasets for wire rope defect recognition, the original wire rope testing signals should be preprocessed through signal denoising and feature extraction. The detailed signal denoising using Haar wavelet transform, signal preprocessing through normalization and multi-dimensional feature extraction are illustrated in this section.

### 3.1. Signal Denoising

According to the signal analysis mentioned above, further signal denoising methods by wavelet transform were studied. The discrete wavelet transform for the time series signal can be described as the inner product of the detection signal and the mother wavelet function, such as,

(5) $$DWT_{x}(m,n)=<s(t),\psi_{m,n}(t)>=2^{-\frac{m}{2}}\int_{R}s(t)\psi (2^{-m}t-n)dt$$

where, *m* and *n* are the scale parameter and shift factor, *s*(*t*) is the wire rope defect signal and *ψ*(*t*) is the mother wavelet function. According to the principle of wavelet threshold denoising method by unbiased estimation, when the decomposition level is set as 12 and the Haar wavelet is chosen, a new time series signal can be obtained through the sorting method for absolute values of *s*(*i)*,

(6) $$f(i)={\left(sort(\left|s(i)\right|)\right)}^{2}\begin{array}{ccc} & \left(i=0,1,2,\ldots ,N-1\right) & \end{array}$$

where *N* is the number of the signal point, and when the denoising threshold of *λ* was chosen as the square root of the new signal series, namely,

(7) $$\lambda =\sqrt{f(i)}\begin{array}{ccc} & \left(i=0,1,\ldots ,N-1\right) & \end{array}$$

The risk of the estimation for the denoising signal can be described as,

(8) $$R(k)=[N-2k+\sum_{i=1}^{k}f(i)+(N-k)f(N-k)]/N$$

When the risk of *R(k)* is the least, the best point of k can be chosen as,

(9) $$k_{\min}=\begin{array}{ccc} \arg & \min & R(k) \end{array}$$

Thus, the threshold of the wavelet denoising function is,

(10) $$\lambda_{k}=\sqrt{f(k_{\min})}$$

**Figure 8:**
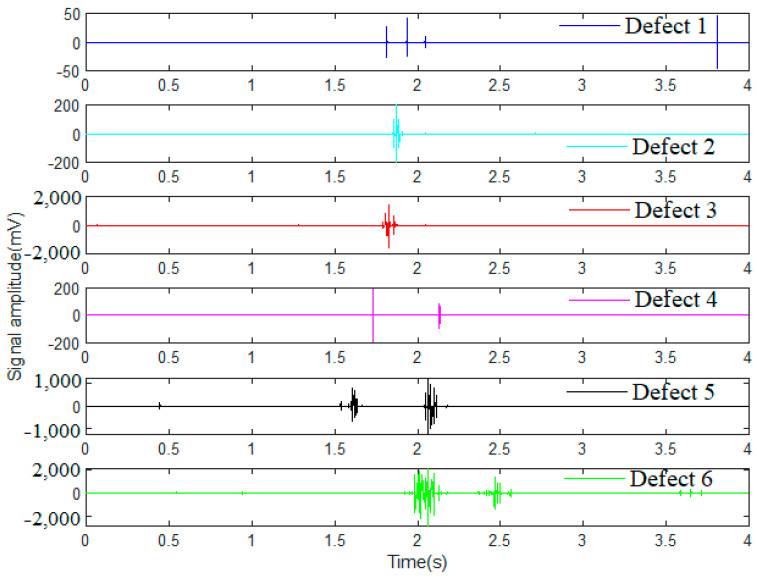
Wavelet denoising signals of different wire rope defects.

After the signal denoising by wavelet transform through Haar function, further differentiated operations between the denoised signal and the original signal were applied. Consequently, typical wire rope defect signals processed from dataset 1 were acquired and are expressed in Figure 8. Compared with the original defect signals expressed in Figure 2a, the final denoised signals all feature prominent and clearer defect waveforms with a few noises, and the wire rope defect signal recognition accuracy and identifiability are greatly improved.

### 3.2. Signal Preprocessing

**Figure 9:**
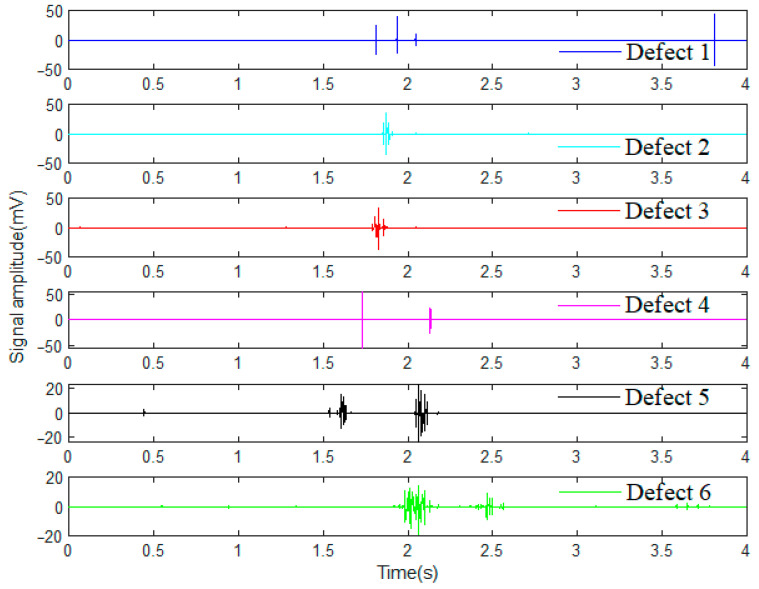
Wire rope defect signals preprocessed by normalization.

To eliminate the influence of the signal data range from different wire rope detection sensors, a normalization method was applied for these datasets, and the Z-score normalization strategy can be described as,

(11) $$S{\left(i\right)}_{norm}=\frac{s(i)-\mu }{\sigma }$$

where *S(i)_norm_* is the normalized time series of the denoised signal *s*(*i*), *μ* and σ are the mean value and standard deviation, respectively. Thus, all different signal datasets can be converted to standard signals obeying the normal distribution with the mean value of 0 and standard deviation of 1. Then, the normalized signals can be applied for training and testing in wire rope defect recognition, and the normalized results for typical wire rope defect signals from dataset 1 are shown in Figure 9. Obviously, the normalized signals are still consistent with denoised signals in amplitude as shown above in Figure 8, meanwhile, the convergence of these datasets can also be guaranteed when the wire rope defect signals are trained and tested by a machining learning classifier.

### 3.3. Feature Extraction

Wire rope detection signals are usually characterized with various features and multiple dimensions, which makes the accurate recognition of defects full of challenges. Therefore, the feature extraction methods by multiple parameters calculations are proposed. Primarily, ten main features for the wire rope signals were extracted, such as the maximum and minimum value, the mean and root mean square (RMS) value, the skewness, kurtosis, and the dynamic range (DR), the crest factor (CF), signal duration as well as the autocorrelation time of the signal data. Specifically, the skewness of γ(*s*), kurtosis of *K*(*s*), dynamic range of Γ(*s*), crest factor of *C*(*s*) can be described and calculated as follows,

(12) $$ϒ(s)=E[{\left(\frac{s-\mu }{\sigma }\right)}^{3}]$$

(13) $$K(s)=E[{\left(\frac{s-\mu }{\sigma }\right)}^{4}]$$

(14) $$\Gamma (s)=20\log10(\frac{\max(\left|s(i)\right|)}{\min(\left|s(i)\right|)})\begin{array}{ccc} & s(i)\neq 0, & i=1,2,\ldots ,N \end{array}$$

(15) $$C(s)=20\log10(\frac{\max(\left|s(i)\right|)}{\sigma })$$

After calculating for the dataset with different wire rope defect detecting signals, typical feature parameters of the normalized dataset 1 were extracted and presented in Table 1 it can be observed that all the RMS values are 1 owing to the signal normalization, and the signal duration is chosen as 4 s to avoid the redundancy.

To verify and compare the validity of the wire rope defect classifier by different recognition methods, six main defects in four different datasets were chosen in the training and testing process, which can be seen in Table 2. In addition, the ten features mentioned above were also chosen in each group of the dataset.

## 4. Proposed Algorithms and Comparisons

Combining the experiments and wire rope defect inspection datasets mentioned above, the proposed new framework of 1D convolution neural network, principles of wire rope recognition method, as well as the comparisons and results are presented in this section, which are expected to demonstrate the feasibility and superior performance of the proposed algorithm.

### 4.1. CNN Framework

**Figure 10:**
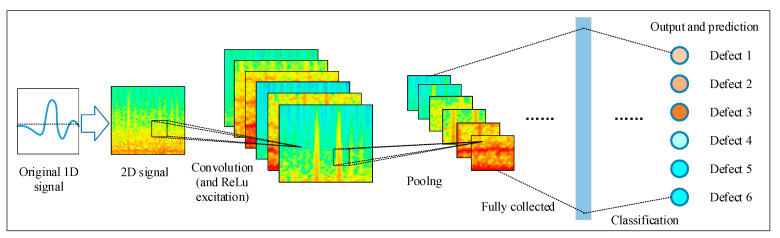
Framework of the 1D-CNNs.

Convolution neural network (CNN) is a multi-layer supervised learning neural network, which has deep hidden layers compared with the common artificial neural network (ANN), and the hidden layers including the commutative convolution layer and pooling layer are the core modules in feature extracting. As shown in Figure 10, the framework of a CNN may be composed of the input layer, convolution layer, excitation layer, pooling layer and fully collected layer. In addition, the stochastic gradient decent with momentum (SGDM) methods are often used in minimizing the loss function and back-adjusting for the weighting parameter. Consequently, the classification accuracy can be improved by multiple training and iteration processes.

The hyperparameters used in the 1D-CNNs is listed in Table 3, for instance,

A typical mean square error function as the loss function in the network evaluation part can be described as,

(16) $$L(\theta )=\frac{\sum_{i=1}^{n}{\left(y^{\left(i\right)}-y^{(i)’}\right)}^{2}}{n}$$

where the *y*^(*i*)^ is the true value and *y*^(*i)’*^ is the predicted value which satisfies the following functional relationship with the input layer of *x*,

(17) $$y^{l}(m,n)=x^{k}(m,n)*g^{kl}(m,n)=\sum_{k=0}^{K-1}\sum_{i=0}^{I-1}\sum_{j=0}^{J-1}x^{k}(m+i,n+j)g^{kl}(i,j)$$

where *g*(*m, n*) represents the convolution kernel, and the size of the convolution kernel is *I* × *J*, *K* is the number of the input channel, while the size of the input matrix is *M* × *N*. Furthermore, the exciting function is usually chosen as the rectified linear function called ReLU, namely,

(18) f(x)=max(0,x)

Compared with other functions such as the sigmoid and tanh, ReLU is featured with faster convergence speed when the SGD method is applied.

### 4.2. 1D-CNNs Recognition Method

**Figure 11:**
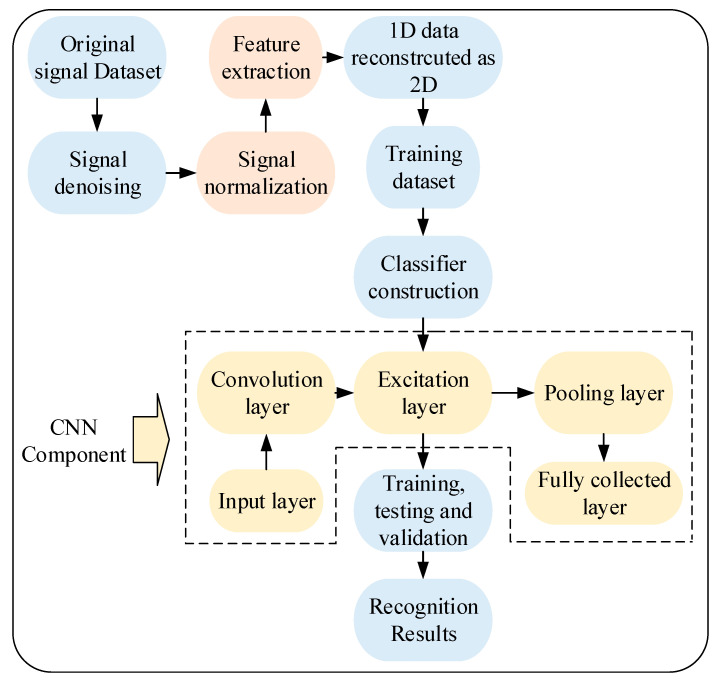
Schematic diagram of the wire rope defect recognition by signal analysis and CNNs.

Specifically, the schematic diagram of wire rope defect recognition by 1D-CNNs classifier is illustrated in Figure 11. Explanatorily, when the 1D original signals acquired from different wire rope defects are denoised and normalized, preliminary features of the signals from different datasets are extracted to establish the sample space for training and testing, where the 1D data are reconstructed as 2D matrix. Then, the defect recognition classifier by CNNs is developed which includes the input layer, convolution layer, excitation layer, pooling layer, and full collected layer as well as the output layer. After multiple iterations of training, testing and validation referring to the classification parameters given in Table 3, the final wire rope defect recognition results can be obtained.

According to the CNN principles and wire rope recognition process, after the 1D original signal reshaping by matrix reconstruction, a 2D dataset can be obtained and applied in the input layer. Concretely, the input layer size is set as 200 × 5 × 1, the pooling method is set as the max pooling, the stride is set as 1, the exciting layer is ReLu, and three fully connected layers with the size of 512,128 and 6 are applied. When the learning rate is 0.01, the iteration number is 200 and the suspensive condition is that the training and testing error is less than 1 × 10^−8^, six output results for different types of wire rope defects can be obtained through the output function of softmax.

### 4.3. Results

**Figure 12:**
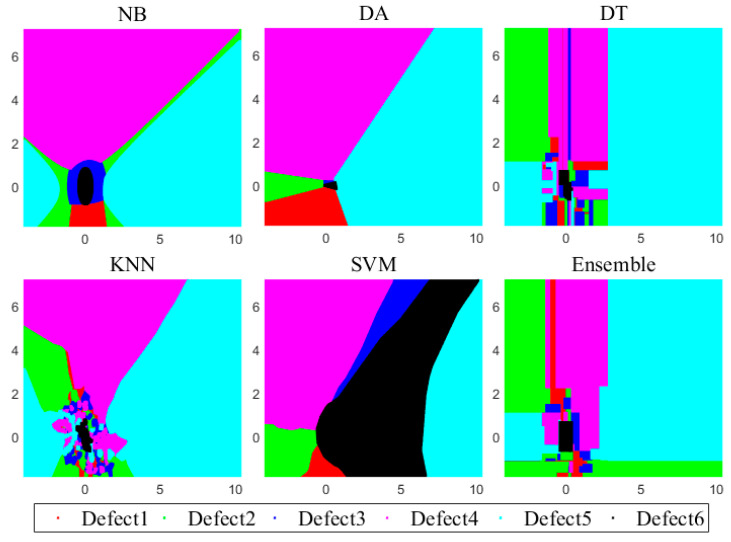
Decision surface by different classifiers.

As intelligent techniques and related research further develop, various machine learning methods and classifiers have been proposed, such as the naive Bayes (NB), discriminant analysis (DA), decision tree (DT) and k-nearest neighbor (KNN) as well as SVM and boost methods. Specifically, 10-fold cross validation was applied for these six machine learning classifiers, where the training and testing proportion were split as 80% and 20%, while the prior probability for each class in NB and DA is empirical. In addition, a linear discriminant method is compared, and the maximal number of decision splits or branch nodes per tree in the DT is 599, the minimum leaf size and parent size is 1 and 10. The number of bins used for every numeric (non-categorical) predictor is set as 50 to speed up the DT training process, where the output tree includes the optimal sequence of pruned subtrees, and the criterion for choosing a split is Gini’s diversity index (GDI). As for the KNN classifier, the nearest neighbors search method is using the exhaustive search algorithm, where each neighbor gets equal weight in the Euclidean distance weight, and the maximum number of data points in the leaf node of the kd-tree is 50. Furthermore, the tolerance for the gradient difference between upper and lower violators obtained by the solver of sequential minimal optimization (SMO), as well as the maximal number of iterations for SMO is 1 × 10^−3^, while the kernel function of radial basis function (RBF) is mainly considered for SVM. The bagging method is mainly applied in the ensemble classifier, and the ensemble learning cycle is 100. When six types of different defects of the wire rope detection datasets mentioned in Table 2 are trained and tested in these classifiers, the decision surface of each classifier is obtained and presented in Figure 12, respectively.

**Figure 13:**
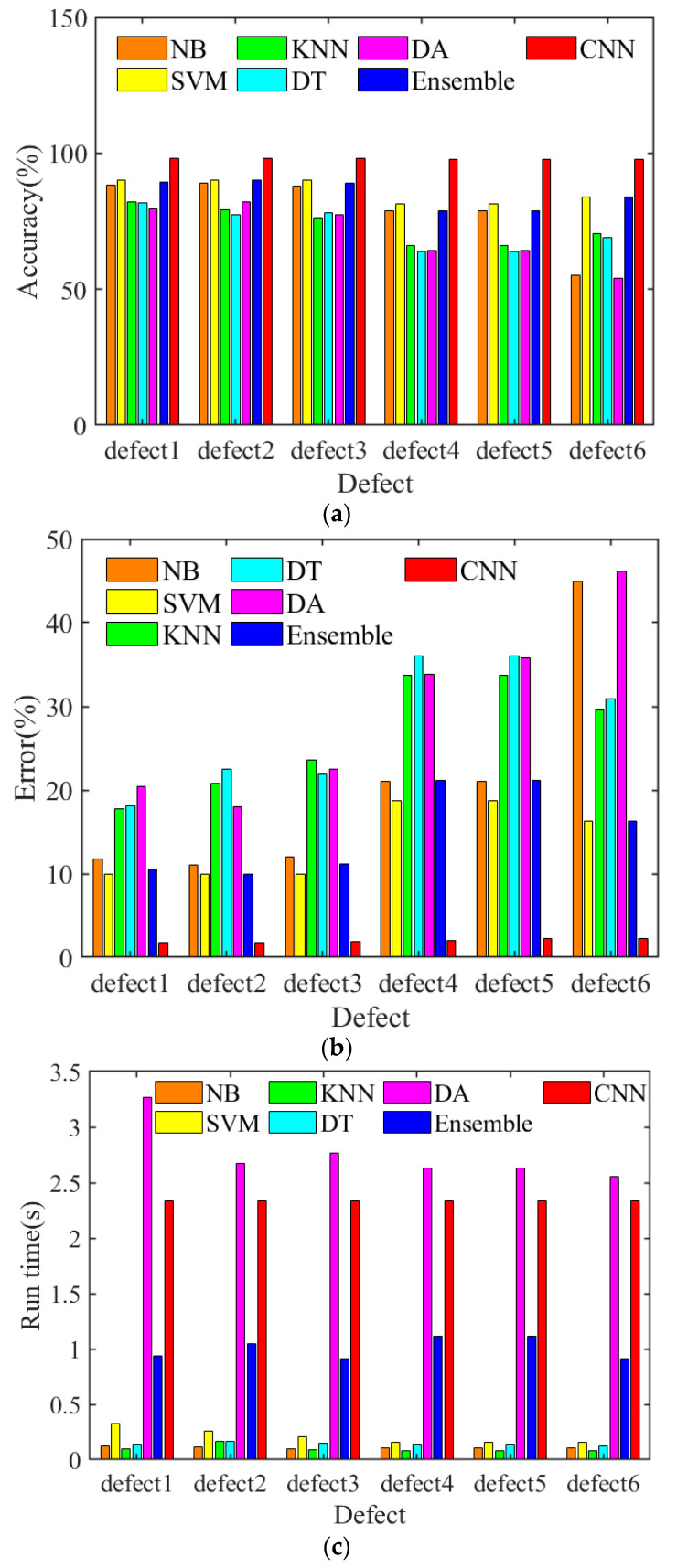
Histograms of the testing and comparisons results: (**a**) testing accuracy for wire rope defects by different classifiers; (**b**) testing error for wire rope defects by different classifiers; and (**c**) run time for wire rope defect recognition by different algorithms.

It can be observed that almost all different defects can be distinguished by these classifiers, especially for classification results via SVM and ensemble learning methods, which are featured with less cross item and erroneous judgement. Similarly, after the training and testing through these machine learning classifiers, defect 1, defect 2 and defect 3 are characterized with higher resolution compared with other types of defects judged by the color of these decision surfaces. On the other hand, comparisons between these common machine learning techniques and the proposed 1D-CNN-based wire rope defect reconstruction and classification methods were also conducted, when the defect recognition performance evaluated through the testing accuracy and error as well as the run time are separately calculated and presented, the comparison results are shown in these histograms in Figure 13.

Explanatorily, after the machine learning training and testing by NB, SVM, KNN, DT, DT, ensemble learning and CNNs classifiers, all the wire rope defects can be well recognized. According to the testing accuracy results shown in Figure 13a, the defect recognition accuracy can reach above 70% through different datasets, while the classification accuracy for defect 6 by NB and DA are relatively lower. In addition, wire rope defect classification accuracies through the classifiers of NB, SVM, and ensemble learning as well as the CNNs are higher than that from KNN, DT and DA. It can also be obviously observed that the testing accuracy is the highest when these defects are classified and recognized by CNNs, which can reach 98.0048%. When the dataset of defects 6 are trained and tested, NB and DA behave the worst in the defect recognition, which featured the highest recognition errors and can be found in Figure 13b. Similarly, the testing and recognition errors for six different types of defects by SVM, ensemble learning and CNNs (1.9952%) are relatively lower than that of the classifiers of KNN, DT and DA. Combining the testing and recognition results from Figure 13a,b, it can be concluded that the wire rope defects classification by the CNNs is the best, and not only can predict and distinguish the six kinds of wire rope defects exactly, but also features the highest recognition accuracy compared with these common machine learning classifiers. However, owing to the deep neural network and complicated convolutional layers, the algorithm run time on the computer through DA and CNNs are also the longest, which were both longer than 2 s when six types of defects from the four datasets were tested, while the run time from the classifiers of NB, SVM, KNN, DT were all lower than 0.5 s, as can be seen in Figure 13c. Namely, although the detection accuracy is guaranteed by the CNNs, the detection efficiency is limited. Further parameters of the mean value, such as the detection accuracy, classification error and run time in evaluating the performance of different classifiers are separately presented in Table 4.

**Figure 14:**
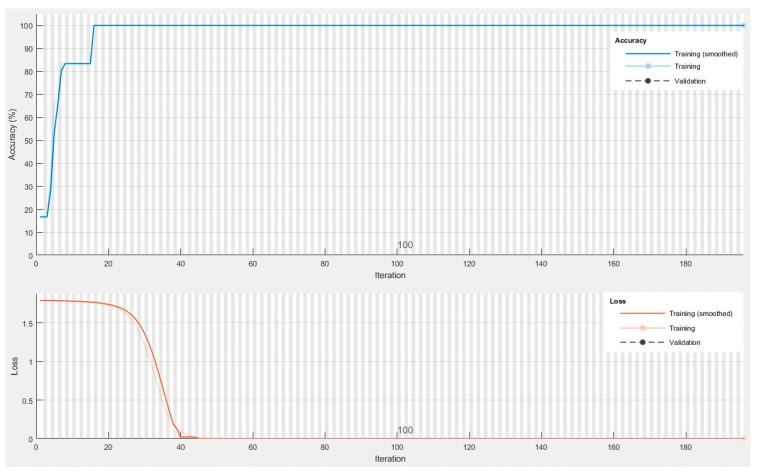
Training and testing curve by CNNs.

Additionally, other related deep learning methods in wire rope defect detection are also presented and compared in Table 5, such as,

According to the testing results shown in Table 4 and the comparison results expressed in Table 5, all the wire rope defect classification mean accuracies are higher than 70% under the testing conditions with a small sample space and common machine learning classifiers, while the recognition accuracy by CNNs can exactly reach 98.0048% for six different defects, and features the best comprehensive defect recognition performance among these methods. Furthermore, the mean run time by the CNNs is relatively shorter than the other combined algorithms, which reached 2.3333 s when the testing and recognition procedures were completed. The details of the testing and training by CNNs are depicted in Figure 14.

Specifically, the accuracy is the highest when the iteration number is bigger than 16, despite the training and validation curves being nearly the same and overlapped. As for the losses results, when the iterations are greater than 40 or 45, the loss is nearly zero in the training and validation procedures. Both results shown in Figure 14 demonstrate that although the training and testing iteration is 200 and the run time is 2.3333 s, most of the time is redundant in the wire rope defect recognition, and only 20 percent of the run time (0.4666 s) is enough for the exact defect classification. Considering the high accuracy of CNNs classifier, it is full of promise in the wire rope defect detection in various application scenes compared with the common machine learning techniques.

## 5. Conclusions and Discussion

Wire rope is one of the most important loading materials in engineering applications, and the defect detection of wire rope is crucial in guaranteeing the safety of human as well as the healthy development of the social economy. However, the sophisticated detection environment and monitoring conditions usually make defect recognition full of difficulty and challenges. Consequently, the proposed MFL signal analysis and 1D-CNN combined defect recognition method not only eliminates the interference of different environment noises, but also improves the defect distinguishing and recognition accuracy, which can reach a mean value of 98.0048%. Compared with common signal processing and defect classification techniques, the proposed combined methods behave better in improving the recognition accuracies for six typical wire rope defects inspection signals from different datasets.

On the other hand, the limitation of the proposed method lies in that the wire rope defect recognition results are influenced by the run time of the algorithms, which may affect the classification efficiency in online or real-time inspection of steel wire ropes. The pooling layer in the CNNs may cause information loss and neglection for the correlation between local and overall network architectures. Therefore, larger numbers of datasets with more defect types and training procedures, and improved pooling structures may decrease the testing and recognition time as well as increase the inspection efficiency. Consequently, future work will include the further optimization, quantitative testing and evaluation for more types of wire rope defects, which can not only provide a reference referring to the discard criteria of wire rope detection, but also guarantee the safety of human life and property. More training and validation of the CNN classification-related algorithms as well as steel wire rope detection and signal processing experiments will also be conducted.

## Figures and Tables

**Table 1:** Feature parameter of Dataset 1.

Features	Data1	Data2	Data3	Data4	Data5	Data6
Max	43.485	35.435	33.356	54.838	23.566	13.759
Min	−43.485	−35.435	−37.403	−54.838	−24.232	−18.706
Mean	1.676 × 10^−18^	−2.031 × 10^−19^	−1.392 × 10^−18^	4.533 × 10^−19^	−4.706 × 10^−18^	−4.912 × 10^−18^
RMS	1	1	1	1	1	1
Skewness	5.287	−2.240 × 10^−8^	−3.212	−0.074	0.361	−1.661
Kurtosis	1365.029	614.899	587.105	2396.053	204.458	89.892
DR	308.961 dB	320.392 dB	324.174 dB	306.019 dB	323.177 dB	348.104 dB
CF	32.767 dB	30.989 dB	31.458 dB	34.782 dB	27.688 dB	25.439 dB
Signal duration	4 s	4 s	4 s	4 s	4 s	4 s
Autocorrelation time	1.998 s	0.033 s	0.049 s	0.402 s	0.488 s	0.472 s

**Table 2:** Wire rope inspection data from four different datasets.

Defect Class	Data Number	Feature Number
Defect 1	8000 × 4	10
Defect 2	8000 × 4	10
Defect 3	8000 × 4	10
Defect 4	8000 × 4	10
Defect 5	8000 × 4	10
Defect 6	8000 × 4	10

**Table 3:** The parameters in the 1D-CNNs architecture.

Name	Parameter	Name	Parameter
Neural network	LeNet-5	Training: testing: validation	6:2:2
Input layer size	200 × 5	Learning rate	0.01
Convolution layer number	4	Mini batch size	128
Padding method	Same padding	Excitation function	ReLu
Pooling method	Average pooling	Max epoch	200
Pooling layer number	1	Soler	SGDM
Fully collected layer	3	Output layer	Softmax

**Table 4:** Comparison of recognition results by different algorithms (mean value).

Method	NB	SVM	KNN	DT	DA	Boost	Our CNNs
Accuracy (%)	79.7083	86.0417	73.4583	72.4375	70.2201	84.9792	98.0048
Error (%)	20.2917	13.9583	26.5417	27.5625	29.4483	15.0208	1.9952
Time (s)	0.1076	0.2107	0.1001	0.1443	2.7526	1.0054	2.3333

**Table 5:** Comparison of wire rope defect recognition results by other related algorithms.

Method	WR-IPDCNN [49]	DL [50]	SVDD [51]	WR-LBPML [52]	RCNN [53]	CDAE-iForest [54]	Our CNNs
Defect type	Surface	Surface	Surface	Surface	Surface	Surface	LF defect
Defect class	2	2	3	2	4	100	6
Accuracy (%)	95.55	99	94	93.3	90.61	93	98.0048
Error (%)	4.45	1	6	6.7	9.39	7	1.9952
Time (s)	22.4	5.74	-	0.014	-	-	2.3333

## Data Availability

The data that supports the finding in this study are not publicly available. Any access request should be sent to liusw@hzau.edu.cn.
